# Protease-activated receptor 2 agonist increases cell proliferation and invasion of human pancreatic cancer cells

**DOI:** 10.3892/etm.2014.2052

**Published:** 2014-11-05

**Authors:** LIQUN XIE, ZEXING DUAN, CAIJU LIU, YANMIN ZHENG, JING ZHOU

**Affiliations:** 1 Department of Gastroenterology, Affiliated Hospital, Logistics University of Chinese People’s Armed Police Forces, Tianjin 300162, P.R. China; 2 Hunan Provincial Corps Hospital, Chinese People’s Armed Police Forces, Changsha, Hunan 410006, P.R. China

**Keywords:** protease-activated receptor 2, trypsin, Ser-Leu-Ile-Gly-Lys-Val, pancreatic cancer, invasion and migration

## Abstract

The aim of this study was to determine the expression of protease-activated receptor 2 (PAR-2) in the human pancreatic cancer cell line SW1990, and to evaluate its effect on cell proliferation and invasion. The expression of PAR-2 protein and mRNA in SW1990 cells was determined by immunocytochemistry and reverse transcription polymerase chain reaction (PCR), respectively. MTT and cell invasion and migration assays, as well as semi-quantitative PCR and zymography analysis, were additionally performed. PAR-2 mRNA was significantly upregulated in the cells treated with trypsin or the PAR-2 activating peptide Ser-Leu-Ile-Gly-Lys-Val (SLIGKV) (P<0.01), but not in the Val-Lys-Gly-Ile-Leu-Ser group (P>0.05). Trypsin and SLIGKV significantly promoted SW1990 cell proliferation in a dose- and time-dependent manner (P<0.05). Compared with the control group, trypsin and SLIGKV significantly increased the mRNA expression (P<0.01) and gelatinolytic activity (P<0.01) of matrix metalloproteinase (MMP)-2. In conclusion, PAR-2 is expressed in SW1990 cells. PAR-2 activation may promote the invasion and migration of human pancreatic cancer cells by increasing MMP-2 expression.

## Introduction

The incidence and mortality rates of pancreatic cancer are similar worldwide (1), and the five-year survival rate of the condition is only 3%. Furthermore, the incidence of early-onset pancreatic cancer is increasing (2). As pancreatic cancer is typically malignant and exhibits a rapid growth rate, metastasis to the lymph nodes and blood occurs frequently, and this is closely associated with the prognosis. Therefore, further study of the potential molecular mechanisms underlying pancreatic cancer cell invasion is of particular significance.

Protease-activated receptor 2 (PAR-2) is a type of G protein-coupled receptor in the cell membrane. PAR-2 expression, which is associated with tumor proliferation, invasion and metastasis, is significantly higher in gastrointestinal cancers, including esophageal, gastric, liver and colorectal, than that in normal tissue cells (3–5). It has been shown that the PAR-2 receptor is expressed in pancreatic cancer tissue (1); however, whether the PAR-2 receptor and its endogenous agonist trypsin is involved in pancreatic cancer metastasis is unknown.

There are four PAR subtypes, known as PAR-1, -2, -3 and -4. Trypsin, tryptase, coagulation factors and other unknown proteolytic enzymes are the natural receptor agonists. Synthetic PAR-2 activation peptides, such as Ser-Leu-Ile-Gly-Arg-Leu, have been widely used in PAR-2 tumor and inflammation research (6). The molecular mechanism study underlying pancreatic cancer invasion and metastasis has become a study focus domestically and abroad.

It has been established that the normal pancreatic acinar and pancreatic cancer cells secrete trypsinogen. Trypsinogen becomes trypsin following activation, and trypsin is a powerful endogenous PAR-2 agonist. Soreide *et al* (7) showed that PAR-2 and trypsin promoted colon cancer invasion and metastasis in association with matrix metalloproteinases (MMPs), and indicated the intrinsic causes of the high malignancy of pancreatic cancer. However, at present the mechanism of PAR-2 in pancreatic cancer invasion and metastasis is unclear. In the present study, the highly invasive and metastatic human pancreatic cancer cell line SW1990 was utilized as the target cells in an attempt to further elucidate the molecular mechanism underlying the invasion and metastasis of pancreatic cancer.

In this study, the human pancreatic adenocarcinoma cell line SW1990 was treated *in vitro* with the anti-PAR-2 agonist peptide (Val-Lys-Gly-Ile-Leu-Ser; VKGILS), trypsin or the PAR-2 agonist (Ser-Leu-Ile-Gly-Lys-Val; SLIGKV). The effects of such treatments on PAR-2 receptor expression levels were determined by reverse transcription-polymerase chain reaction (RT-PCR) and immunocytochemistry methodology. The effect of the activated PAR-2 receptor agonist on SW1990 cell invasion and metastasis was also investigated for its possible use as a novel cancer drug candidate for clinical application in the future.

## Materials and methods

### Materials

RPMI-1640 medium was purchased from Gibco-BRL (Grand Island, NY, USA). Fetal bovine serum (FBS) was obtained from the Institute of Hematology, Chinese Academy of Medical Sciences (Tianjin, China). Polyclonal PAR-2 antibody was purchased from Santa Cruz Biotechnology, Inc. (Santa Cruz, CA, USA). Streptomycin avidin-peroxidase immunohistochemistry and diaminobenzidine (DAB) color kits were obtained from Fuzhou Maixin Biotechnology Development Co., Ltd. (Fuzhou, China). TRIzol^®^ was purchased from Invitrogen Life Technologies (Carlsbad, CA, USA). PCR marker and RT-PCR kits were obtained from Dalian Bao Biological Engineering Co. (Dalian, China). The PAR-2 agonist (SLIGKV) and anti-PAR-2 agonist (VKGILS) peptides were synthesized by Meilian (Xi’an) Biological Technology Co., Ltd. (Xi’an, China). Matrigel™ glue was purchased from BD Biosciences (Bedford, MA, USA). The Transwell^®^ chamber was purchased from Millipore (Billerica, MA, USA). PAR-2, MMP-2 and MMP-9 primers were synthesized by Invitrogen Life Technologies.

### Cell culture and experimental grouping

FBS RPMI-1640 complete medium with 100 ml/l FBS was applied and the cells were incubated in a 50-ml/l CO_2_ incubator at 37°C with a relative humidity of 95%. When the cells covered 70–80% of the bottle bottom, they were digested by 0.25% trypsin and 0.03% EDTA. Cells in the logarithmic growth phase were utilized for the subsequent experiments. In the MTT experiment, there were four groups: Control (with medium only), trypsin (at concentrations of 0.1, 1, 10 and 100 nM), SLIGKV (at concentrations of 5, 25, 50 and 100 μM) and the VKGILS-NH_2_ group (at concentrations of 5, 25, 50 and 100 μM). In the RT-PCR, cell migration and invasion test and gelatin zymography experiment, the cells were divided into the control, VKGILS-NH_2_ (50 μM), trypsin (10 nM) and SLIGKV (50 μM) groups. Prior to treatment, cells were cultured in serum-free RPMI-1640 for 24 h for cell cycle synchronization. The present study was approved by the ethics review board of the Logistics University of Chinese People’s Armed Police Force (Tianjin, China).

### Immunocytochemical detection

In each well of the six-well plate, 1×10^5^ cells were inoculated at 37°C for 24 h. When cell fusion reached ~60%, the coverslip was removed and the cells were fixed with ice-cold methanol-acetone (1:1). Following the removal of endogenous hydrogen peroxide enzyme by 3% hydrogen peroxide, the cells were preserved in normal goat serum at room temperature for 10 min. The 1:100 diluted goat PAR-2 polyclonal antibody was added (replaced with phosphate-buffered saline in the negative control) and the cells were incubated overnight at 4°C. The biotin-labeled secondary antibody was then added and the solution was incubated at 37°C for 10 min. Following incubation, horseradish peroxidase-labeled streptomycin avidin complex was added and the cells were further incubated for 10 min at 37°C, prior to coloration by DAB and hematoxylin. The cells were observed under the microscope (Olympus CK-2; Olympus Corporation, Tokyo, Japan) and images were captured.

### RT-PCR

Total RNA was extracted from the SW1990 cells by TRIzol reagent. The integrity of RNA was identified by electrophoresis of 1% agarose gel (the ratio of 28S and 18S RNA band was ≥2). Total RNA (1 μg) was reverse transcribed in the following conditions: 10 min at 30°C, 30 min at 42°C, 5 min at 99°C and 5 min at 5°C. β-actin was used as the internal control. The primers used in this study are provided in Table I. Following the electrophoresis of 2% agarose gel, PCR products were scanned by the gel automatic imaging system. Relative value analysis was determined by β-actin correction and the value was expressed as the ratio of the absorbance of the detected band to the absorbance of the β-actin band.

### MTT assay

The cell suspension was incubated in a 96-well plate with a density of 6×10^4^/ml. Following culture at 37°C for 24 h, the medium was replaced with serum-free medium and cell suspension was continued to culture for 24 h. The suspension was dosed according to each group with eight wells, respectively. A total of 20 μl MTT solution (5 g/l) was added in each well after 48 h of culture. Four hours later, the culture was terminated and the culture medium was removed. A total of 150 μl dimethyl sulfoxide was added to each well, prior to oscillation for 30 min at room temperature for the crystals to dissolve. An absorbance value for each well at 490 nm wavelength was determined by the enzyme mark instrument (Model 550; Bio-Rad Laboratories Inc., Hercules, CA, USA). This experiment was repeated three times.

### Cell migration and invasion assay

The method for the cell migration and invasion assay was performed according to that described previously for the Albini’s chamber test (8) with certain modifications. Briefly, SW1990 cells in the logarithmic growth phase were preserved in serum-free RPMI-1640 medium for 24 h. Following digestion by 0.04% EDTA, the single-cell suspension was made by serum-free RPMI 1640 medium. The cell concentration was adjusted to 2.5×10^5^/ml and cell viability was detected by trypan blue staining. Cell suspension (0.2 ml) was added to each upper chamber of the Millicell^®^ chamber room, and drugs were added according to the experimental group with three wells in each group. In the lower chamber, 600 μl cell culture medium RPMI-1640 containing 10% fetal calf serum was added in each hole. The suspension was cultured in the culture box for 24 h, and the microporous membrane was removed. Cells that did not enter the membrane were wiped by a cotton ball, and the remaining cells were fixed and stained by methanol and hematoxylin. The number of cells through the membrane was counted in five high-power fields (magnification, ×400) under the microscope, and the average number was calculated. In the cell invasion assay, the Matrigel matrix gel was melted at 4°C in advance. Each microcellular polycarbonate membrane surface was paved with 40 μl diluted Matrigel glue ratio of Matrigel and serum-free medium, 1:4). The cells were solidified into the culture box for 4 h and cell concentration was adjusted to 1×10^5^/ml. The remaining steps were the same as those for the migration test.

### Gelatin zymography

Total cellular protein was extracted using protein extraction reagent [50 mM Tris (pH 8.0), 1% NP-40, 0.1% sodium dodecyl sulfate, 0.02% sodium azide, 150 mM sodium chloride and 0.5% phenylmethylsulfonyl fluoride]. Sample protein content was determined and adjusted using the Bicinchoninic Acid Protein Quantitation kit (Beyotime Institute of Biotechnology, Haimen, China) and electrophoresed by 10% polyacrylamide gel electrophoresis gel containing 1 g/l gelatin. The sample was agitated and washed in the eluent (2.5% Triton X-100, 50 mM Tris-HCl, 5 mM CaCl_2_ and 1 μM ZnCl_2_, pH 7.0) at room temperature for 15 min three times and then incubated at 37°C overnight in gelatinase buffer [50 mM Tris-HCl (pH 7.4), 200 mM NaCl and 5 mM CaCl_2_). The protein was fixed and stained for 30 min respectively in 1 g/l Coomassie brilliant blue (concentration ratio of acetic acid:methanol:water, 1:4:5) at room temperature. The protein was destained by eluent excluding Coomassie brilliant blue and a clear strip appeared. The active sites of the protease were bright and transparent. Strip area, width and gray value was detected by a gel automatic imaging system (GDS-8000; Ultra Violet Products Inc., Upland, CA, USA), and the relative value of MMP-2 and MMP-9 was calculated based on the gray values of the deeply-stained proteins.

### Statistical analysis

Statistical analysis was performed using SPSS 13.0 statistical software (SPSS, Inc., Chicago, IL, USA). Measurement data are expressed as the mean ± standard deviation. Comparisons between groups were conducted using the Student’s t-test or Wilcoxon rank sum test. P<0.05 was considered to indicate a statistically significant difference, and P<0.01 was considered to indicate a highly significant difference.

## Results

### PAR-2 protein is expressed mainly in the cell membrane and cytoplasm of SW1990 cells

**Figure 1:**
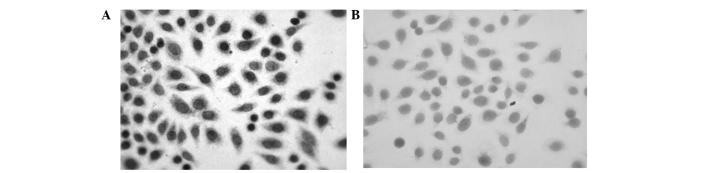
Immunohistochemical detection of PAR-2 expression in SW1990 cells (magnification, ×400). (A) Positive expression of PAR-2 in SW1990 cells using goat anti-PAR-2 antibody. (B) Negative control (no antibody was used). PAR-2, protease-activated receptor 2.

To investigate the expression of PAR-2 protein in SW1990 cells, immunocytochemical detection was performed. As shown in Fig. 1, PAR-2 protein expression, shown by brown-yellow staining, was observed predominantly in the cell membrane and cytoplasm. In the negative control group, the cell membrane and cytoplasm were not stained. These results showed that PAR-2 is expressed mainly in the cell membrane and cytoplasm of SW1990 cells.

### SLIGKV and trypsin increase PAR-2 mRNA expression in SW1990 cells

The anti-PAR-2 agonist peptide (VKGILS) and the PAR-2 agonist (SLIGKV) were used in this study. The cells were treated with VKGILS-NH_2_ (50 μM), SLIGKV (50 μM) or trypsin (10 nM) for 18 h, or were left untreated (medium only). To investigate the effects of the treatments on the expression of PAR-2 mRNA in SW1990 cells, RT-PCR was performed. β-actin served as the internal control.

**Figure 2:**
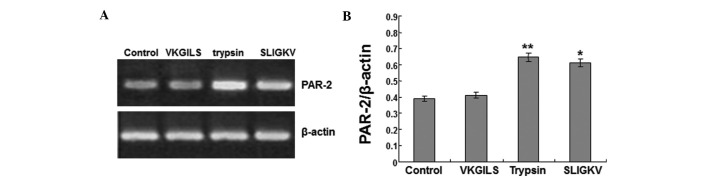
PAR-2 mRNA expression in SW1990 cells by RT-PCR detection. The cells were treated with VKGILS (50 μM), SLIGKV (50 μM) or trypsin (10 nM) for 18 h, or were not treated (medium only). RT-PCR was performed. β-actin served as the internal control. (A) A representative image is shown. (B) Data from at least three repeated RT-PCR experiments. ^**^P<0.01 and ^*^P<0.05 vs. the control group. RT-PCR, reverse transcription-polymerase chain reaction; VKGILS, Val-Lys-Gly-Ile-Leu-Ser; SLIGKV, Ser-Leu-Ile-Gly-Lys-Val; PAR-2, protease-activated receptor 2.

As shown in Fig. 2A and B, the PAR-2 mRNA expression level in the VKGILS group (0.412±0.036) was similar to that in the control group (0.391±0.022). No significant difference in expression was identified between the control and VKGILS groups. However, in the trypsin and SLIGKV groups, PAR-2 mRNA expression levels were increased to 0.645±0.038 and 0.612±0.042, respectively, and were significantly higher than the PAR-2 mRNA levels in the control group (0.391±0.022) (P<0.05). Therefore, these results suggest that SLIGKV and trypsin treatments increase PAR-2 mRNA expression in SW1990 cells.

### Trypsin and SLIGKV increase SW1990 cell proliferation

**Figure 3:**
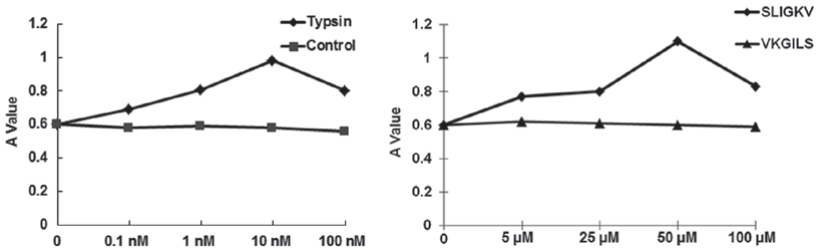
Effect of VKGILS, trypsin and SLIGKV on SW1990 cell proliferation. The SW1990 cells were treated with trypsin, SLIGKV and VKGILS at the concentrations as indicated. The cells treated with medium were used as the control. MTT assay was performed. MTT solution (20μl; 5 g/l) was added to each well after 48 h of culture. Each well was then supplemented with 150 μl dimethyl sulfoxide and oscillated for 30 min at room temperature for the crystals to dissolve. The absorbance (A) value for each well at 490 nm wavelength was determined. This experiment was repeated three times. VKGILS, Val-Lys-Gly-Ile-Leu-Ser; SLIGKV, Ser-Leu-Ile-Gly-Lys-Val.

To determine if treatment with VKGILS, trypsin or SLIGKV affected SW1990 cell proliferation, the MTT assay was performed. As shown in Fig. 3, VKGILS slightly affected cell proliferation, without a significant difference when compared with the control group (P>0.05). However, trypsin and SLIGKV increased the proliferation of pancreatic cancer SW1990 cells in a concentration-dependent manner when compared with the control group (P<0.05). Treatments with high concentrations of trypsin (100 nM) and SLIGKV (100 μM) were harmful to cell proliferation, as expected. These results suggest that trypsin and SLIGKV increase SW1990 cell proliferation.

### SW1990 cell invasion and migration are increased by trypsin or SLIGKV

**Figure 4:**
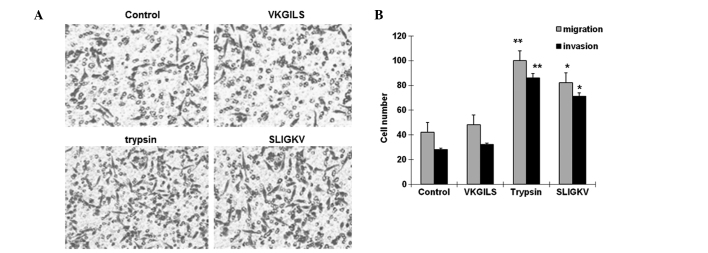
Effect of trypsin, SLIGKV and VKGILS on SW1990 cell migration and invasion determined by the Transwell^®^ chamber method (inverted microscope; magnification, ×200). The cells were treated with VKGILS-NH_2_ (50 μM), SLIGKV (50 μM) or trypsin (10 nM) for 24 h, or were not treated (medium only). (A) Representative images. (B) Data from at least three repeated experiments. ^**^P<0.01 and ^*^P<0.05 vs. the control group. VKGILS, Val-Lys-Gly-Ile-Leu-Ser; SLIGKV, Ser-Leu-Ile-Gly-Lys-Val.

To determine if treatment with VKGILS, trypsin or SLIGKV affected the invasion and migration of SW1990 cells, the cells were treated with VKGILS (50 μM), SLIGKV (50 μM) or trypsin (10 nM) for 24 h, or left untreated (medium only). Cell migration and invasion assays were subsequently performed. As shown in Fig. 4A and B, in these cell migration tests, SW1990 cells disrupted the Matrigel matrix. The number of cells penetrating the microporous membranes in the trypsin (100.8±12.9) and SLIGKV (89.6±10.9) groups was significantly higher than the number in the control (43.3±9.2) and the VKGILS (50.1±7.5) groups. In the invasion tests, SW1990 cells degraded the Matrigel matrix. The number of cells that penetrated the microporous membrane in the trypsin (81.5±8.1) and SLIGKV (71.7±5.2) groups was significantly higher than the number in the control (28.3±5.6) and the VKGILS (32.2±7.8) groups. These results suggest that SW1990 cell invasion and migration were increased by trypsin or SLIGKV when compared with the control and VKGILS treatments.

### Changes in mRNA expression and enzymatic activity of MMP-2 and MMP-9

To investigate the mechanisms underlying the effects of trypsin or SLIGKV on SW1990 cell invasion and migration, the cells were treated with VKGILS (50 μM), trypsin (10 nM) or SLIGKV (50 μM) for 24 h, or were left untreated (medium only). The mRNA expression and enzymatic activity changes of MMP-2 and MMP-9 were determined.

**Figure 5:**
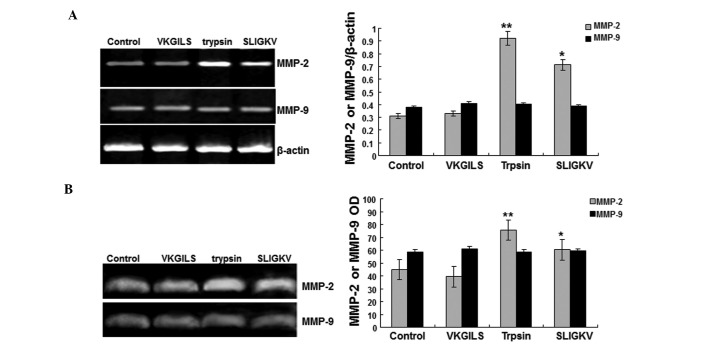
Effect of VKGILS, trypsin and SLIGKV on MMP-2 and MMP-9 expression. The cells were treated with VKGILS-NH_2_ (50 μM), SLIGKV (50 μM) or trypsin (10 nM) for 24 h, or were not treated (medium only). (A) Reverse transcription-polymerase chain reaction detection. (B) Activity measurements of MMP-2 and MMP-9 enzymes by gelatin zymography detection. ^**^P<0.01 and ^*^P<0.05 vs. the control group. VKGILS, Val-Lys-Gly-Ile-Leu-Ser; SLIGKV, Ser-Leu-Ile-Gly-Lys-Val; MMP, matrix metalloproteinase; OD, optical density.

The RT-PCR results (Fig. 5A) showed that VKGILS had no detectable effects on MMP-2 and MMP-9 mRNA levels when compared with the control group. However, the MMP-2 mRNA levels in the trypsin (0.921±0.032) and the SLIGKV (0.712±0.024) groups were significantly higher than those in the control (0.310±0.038) and VKGILS (0.378±0.029) groups. No significant differences were identified in the MMP-9 mRNA levels among the four groups (P>0.05) (Fig. 5A). These results suggest that trypsin and SLIGKV increase the expression levels of MMP-2, but not MMP-9.

Gelatin zymography results (Fig. 5B) showed that MMP-2 enzyme activity in the trypsin (75.6±6.1) and SLIGKV (60.4±4.6) groups was significantly higher than that in the control (44.9±4.2) and VKGILS (39.3±5.2) groups. The MMP-9 enzyme activity was not significantly changed (Fig. 5B). These results suggest that trypsin and SLIGKV increase the activity of MMP-2, but not MMP-9.

## Discussion

In this study, through immunocytochemistry and RT-PCR, it was found that PAR-2 is expressed at the gene and protein levels in the human pancreatic cancer cell line SW1990. In the trypsin and synthetic PAR-2 agonist peptide SLIGKV groups, the PAR-2 mRNA expression level was increased compared with that in the control and inverse agonist peptide groups. This indicated that PAR-2 was present in the human pancreatic cancer cell SW1990 and that its expression increased following activation. In addition, the results suggested that the PAR-2 agonists activated and upregulated PAR-2 levels, thus promoting cancer cell proliferation, suggesting that PAR-2 activation may be crucial in the development of pancreatic cancer. At certain concentrations and time ranges, trypsin and the PAR-2 agonist SLIGKV were found to significantly promote SW1990 proliferation in a concentration- and time-dependent manner. This is consistent with the theory that PAR-2 promotes colon, gastric, breast, liver, esophageal and lung cancer cell proliferation (9). It is suggested that this cell proliferation is associated with the MMP-epidermal growth factor receptor-mitogen-activated protein kinase-extracellular signal-regulated kinase 1/2 (MMP-EGFR-MAPK-ERK1/2) pathway, and that PAR-2 promoted colon and gastric cancer cell proliferation and invasion through the MMP-EGFR-MAPK-ERK1/2 pathway following activation. It is additionally suggested that PAR-2 activated the Ca^2+^ channel through the activation of the MAPK-ERK1/2 pathway to promote prostaglandin E2 release, and then activated EGFR to promote cell proliferation (3–5,10). It can be inferred that PAR-2 may also promote cancer cell proliferation subsequent to activation through the above pathway.

Invasion and metastasis are characteristic of malignant tumors. Two key steps are involved in metastasis: The degradation of the basement membrane and extracellular matrix, which allows breakthrough into the blood circulation or target organ and new vessel formation subsequent to entering the target organs so that metastatic lesions can increase rapidly (11,12). The damage to the integrity of the basal cell membrane is the important sign for malignant tumor invasion. In this preliminary study, it was investigated whether the activation of PAR-2 promotes pancreatic cancer invasion and metastasis in these two key steps. The Transwell chamber was applied to stimulate the extracellular matrix microenvironment, and the effect of PAR-2 activation on the invasion and migration of the pancreatic cancer cell SW1990 was observed. It was found that trypsin and the PAR-2 activating peptide significantly enhanced SW1990 pancreatic cancer cell invasion and migration, showing that PAR-2 activation is associated with pancreatic cancer invasion. Furthermore, it was found that the invasion and migration of SW1990 cells was higher in the trypsin group than that in SLIGKV group. This was consistent with the above-mentioned previous studies showing that trypsin promotes colon cancer invasion and metastasis through its close association with MMPs, and that trypsin can also be activated by PAR-2.

Soreide *et al* (7) showed that colon cancer metastasis is closely associated with the MMP family. MMP-2 and MMP-9 are the key members of the MMP family, and they are closely associated with tumor metastasis since they can degrade the extracellular matrix and disrupt the basement membrane. The association between MMPs and pancreatic cancer metastasis has become a study focus. Bramhall *et al* (13) found through the immunohistochemical method that the expression level of MMP-2 was significantly higher in pancreatic cancer tissue than that in other tissues, and MMP-2 expression was significantly higher in tumor cells than that in interstitial cells. Koshiba *et al* (14) further showed through gelatin zymography and western blot analysis that MMP-2 was associated with pancreatic cancer progression. MMP-2 protein expression in pancreatic carcinoma specimens with distant and lymph node metastasis was significantly higher than that in specimens without metastasis, indicating that MMP-2 level is associated with pancreatic adenocarcinoma invasion, metastasis and prognosis. In the present study, the effects of PAR-2 activation on the gene expression and gelatinase activity of MMP-2 and MMP-9 were determined through RT-PCR and gelatin zymography assay. It was found that, in the trypsin and PAR-2 agonist groups, MMP-2 mRNA level and gelatinase activity were increased, which was consistent with the aforementioned study findings. However, there was no clear association between the gene expression and gelatinase activity of MMP-2 following PAR-2 activation. It has been shown that tumor necrosis factor-α induces MMP expression through MAPK signaling system-mediated nuclear factor-κB activation (15), and PAR-2 promotes numerous types of tumor cell proliferation through the MAPK signaling pathways (16). Therefore, it is hypothesized that PAR-2-induced MMP-2 generation may also share this pathway; this will be a further study focus.

In conclusion, in this study it was found that PAR-2 expressed in the pancreatic cancer cell line SW1990 exhibits an important function in SW1990 cell proliferation and invasion following its activation. Its mechanism may be associated with the PAR-2/MMP-2 pathways. Thus this pathway may be an important target for the prevention and treatment of pancreatic cancer. Further studies of the dynamic expression of PAR-2 and tumor metastasis and the associated signal transduction pathways are required.

## Figures and Tables

**Table I:** Primers used in this study.

Primer	Sequence	Product (bp)
PAR-2_FP	5′-AGAAGCCTTATTGGTAAGGTT-3′	582
PAR-2_RP	5′-AACATCATGACAGGTCGTGAT-3′	
MMP-2_FP	5′-CAGGCTCTTCTCCTTTCACAAC-3′	398
MMP-2_RP	5′-AAGCCACGGCTTGGTTTTCCTC-3′	
MMP-9_FP	5′-TCCCCTACGTCACCTATGACAT-3′	172
MMP-9_RP	5′-GCCCAGCCCACCTCCACTCCTC-3′	
β-actin_FP	5′-TGTTTGAGACCTTCAACACCC-3′	540
β-actin_RP	5′-AGCACTGTGTTGGCGTACAGG-3′	

PAR-2, protease-activated receptor 2; MMP-2, matrix metalloproteinase 2; FP, forward primer; RP, reverse primer.
